# The process of racial discrimination: towards a unified theory

**DOI:** 10.3389/fsoc.2026.1748904

**Published:** 2026-08-11

**Authors:** Anaïd Lindemann

**Affiliations:** Institute for Social Sciences of Religions (ISSR), University of Lausanne, Lausanne, Switzerland

**Keywords:** intergroup relations, prejudice, process model, racial discrimination, stereotypes, racism, responses to discrimination, stigmatization

## Abstract

Scholars in the field of intergroup relations agree that discrimination is a dynamic process with causes and consequences. However, the way in which related concepts—such as stereotype, prejudice, or stigmatization—shape this process has never been integrated together in a unified theory. This paper aims to map and describe this process theoretically by showing how these concepts are interrelated. I do so by drawing on seminal works in the field and by linking together middle-range theories that have empirical support. The result is a five-part process model of discrimination that integrates the multidimensionality of discrimination and three perspectives from which discrimination can be studied. This unified theory not only serves pedagogical purposes by providing a synthesized overview of the major theories in the field but also represents a heuristic tool for empirical research on the production of disadvantages in racialized contexts.

## Introduction

“Discrimination,” “prejudice,” “stigmatization,” “stereotypes”—these terms are often used interchangeably in everyday language. In the social sciences, however, they are concepts with specific meanings and definitions. While many handbooks and scholarly publications rightly treat these concepts separately, they often fail to show how they are interrelated: it is not uncommon to read a chapter on “prejudice and discrimination” that goes through definitions and operationalizations of these theoretical categories one by one, without ending with a model that integrates them (see, for example, Dovidio et al., 2005, Pettigrew, 2015, Quillian, 2006). On the other hand, it is well established that racial discrimination is a process, and that it has causes and consequences. Therefore, at least in theory, it should be possible to map this process in an overarching theory, from causes to consequences.

Existing models that attempt to map the full process of racial discrimination remain unsatisfactory, as they either omit key mechanisms or lack analytical clarity (Feagin and Eckberg, 1980; Williams, 2023). As a result, convincing general models of discrimination are lacking, a gap this paper seeks to address by developing a unified theoretical framework that integrates key concepts and middle-range theories into a coherent model.

This paper focuses on racial discrimination. While early sociological uses of the concept of race referred primarily to groups defined through biological or phenotypical criteria, contemporary scholarship emphasizes that race is socially constructed, historically contingent and “unreliable as supposed boundaries shift” (Omi and Winant, 2014) over time. Hence, research on racialization shows that racial meanings can, under specific social and historical conditions, be extended to groups defined in ethnic, cultural, or religious terms (Miles and Brown, 2003; Carr, 2016; Banton, 2018). Unless otherwise specified, references to discrimination in what follows should therefore be understood as referring to racial discrimination.

Discrimination is a particularly complex social phenomenon to study: it is considered “multifaceted” (Allport, 1954 (1979)), “a moving target” (Massey, 2005), “extremely difficult to study” (Pettigrew, 2015), difficult to measure (Quillian, 2006), and involves “conceptual complexity” (Pettigrew and Taylor, 2015). Indeed, discrimination can be studied from a variety of perspectives and at different points in a process: when a scholar studies discrimination, they may examine the impact of a political environment on the population’s attitudes toward outgroups (Helbling and Traunmüller, 2016), or the distribution of those attitudes in the majority population (Strabac and Listhaug, 2008), or how members of a minority group perceive discrimination (Diehl et al., 2021), or how it affects their economic opportunities (Zwysen et al., 2021), or even how minority groups respond to it (Lalonde and Cameron, 1994). Since Allport (1954)(1979) seminal work, which was surprisingly general, research on discrimination has specialized and focused on very specific aspects of discrimination (Ribes, 2017; Casadevall and Fang, 2014; Leahey, 2007). While empirical studies must necessarily focus on one aspect of discrimination, the bigger picture of discrimination is somewhat difficult to grasp.

To provide a unified and comprehensive account of this process, I draw on established theories from multiple disciplines (primarily psychology, social psychology, economics and sociology) developed by foundational scholars in the study of discrimination. Given the theoretical scope of this paper, an interdisciplinary approach is essential, yet it also complicates the selection of relevant theories and authors. Criteria for inclusion in this attempt at a unified theory of racial discrimination are twofold: first, the extent to which a scholar or study has influenced the field, as indicated by extensive citation; and second, the presence of empirical support for the theoretical framework under consideration.

Like any general model or theory, the framework proposed here is not intended for direct empirical testing, as only middle-range theories and their derived hypotheses are subject to empirical validation (Merton, 2007). Instead, this conceptual model integrates middle-range theories into a unified theory of racial discrimination. The advantages of this approach are manifold. First, by clarifying the distinctions and interconnections among key concepts, this process-based model enables scholars to precisely situate their contributions within the broader literature on discrimination or within specific subfields (e.g., labor market discrimination, attitudinal studies, responses to racism). Second, it facilitates the generation of precise hypotheses regarding specific aspects of the discrimination process and the relationships between them (e.g., how attitudes translate into discriminatory behaviors, the conditions under which different responses to discrimination emerge, or the effects of these responses on life chances). Third, it serves pedagogical purposes, both by synthesizing key concepts in a single framework and by offering a structured tool for teaching discrimination studies, aiding students in grasping the diverse methodological and theoretical approaches used in the field.

**Figure 1:**
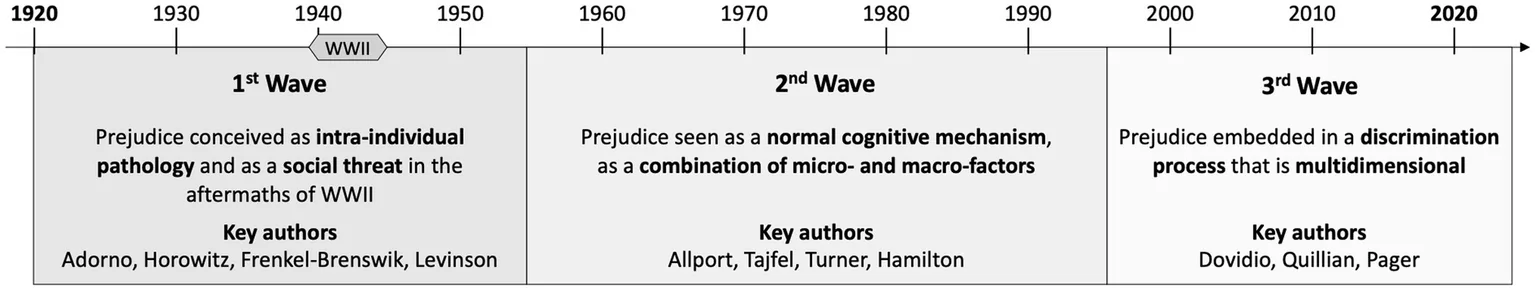
Timeline of the evolution of the scholarly research on racial prejudice and discrimination, based on Kaiser and Miller’s (2001) work.

**Figure 2:**
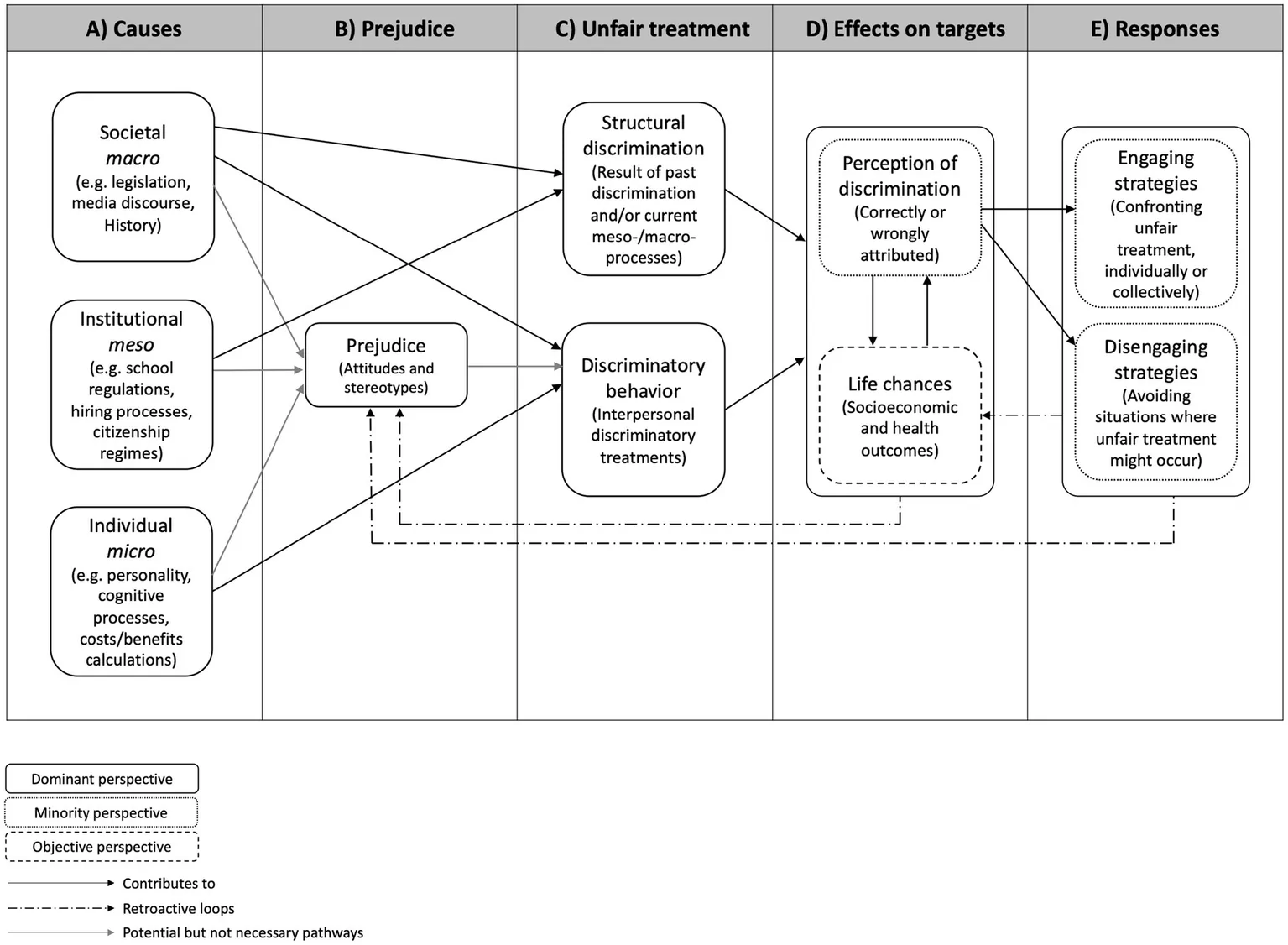
Model of the process of discrimination from causes to consequences, with perspectives and retroactive loops.

The paper is structured as follows: First, the background section provides a concise overview of the foundations of discrimination research and how subsequent theoretical approaches have expanded its scope (Figure 1), providing key concepts later incorporated into the model. Next, the unified theory is developed step by step (A to E), with a visualization of the complete model (Figure 2). The final section synthesizes the discussion and demonstrates how the model can be applied for both scientific inquiry and pedagogical purposes. Lastly, the conclusion addresses the model’s key advantages and limitations.

## Background

### Allport: early milestones

Social scientists usually refer to Allport’s seminal 1954 book, *The Nature of Prejudice*, as the cornerstone of the study of prejudice^1^. His book includes concepts with definitions that are still used today by scientists who study the phenomena of discrimination: “The table of contents […] has in fact organized the scholarly study of the important concept of prejudice. The Nature of Prejudice delineated the area of study, set up its basic categories and problems, and cast it in a broad, eclectic framework that remains today” (Allport, 1954 (1979)). Indeed, what strikes today’s reader is the very broad range of concepts linked to discrimination covered in a single book: prejudice, discriminatory behavior, effects on victims, effects on the majority population, influence of legislation, anti-racism agencies, and so on.

With this general but very well-documented book, Allport laid the foundation for a discipline that would only expand over the decades: Dovidio and his colleagues (Dovidio et al., 2010) find that since 1965, the proportion of articles in leading social psychology journals dealing with prejudice or stereotyping has increased nearly tenfold. Moreover, Allport’s work represents a critical turning point in which prejudice is conceived as a normal cognitive mechanism rather than a pathological anomaly: according to him, ethnic prejudice is widespread simply because the cognitive processes that lead to prejudice are based on natural functions of the mind (Allport, 1954 (1979)).

According to Dovidio (2001), research on prejudice followed three general waves throughout the 20th century (Figure 1). During the first wave, which spanned from the 1920s to the 1950s, scholars conceived of prejudice as a pathological disruption of cognitive processes and, in the aftermath of World War II, as a threatening social problem. This paradigm led researchers to focus on individual-level factors such as personality traits (Adorno et al., 1950) and family history (Frenkel-Brunswik, 1948; Horowitz and Horowitz, 1938).

With Allport, a second wave opened the door to a new conceptualization of prejudice as a normal function of the human mind but influenced by social factors such as norms, ideologies, or social structures. During this wave, micro- and macro-level factors causing discrimination were studied: while Hamilton (1981) showed that stereotyping aims to reduce the otherwise indigestible complexity of information for an individual, Tajfel and Turner (1979) showed how social identity, i.e., the sense of belonging to social groups, can generate intergroup antagonism and discrimination.

The third wave, which began in the mid-1990s, was characterized by the need to approach prejudice as multidimensional, at both the intrapersonal and interpersonal levels: implicit (unconscious) bias and subtle forms of prejudice were studied through experiments with new technologies that made them possible (Greenwald et al., 1998) and the effects of prejudice on targeted individuals began to be studied more closely, rather than focusing only on prejudiced individuals.

These historical developments in scholarship led to new approaches to discrimination that called for a modeling of discrimination that integrated these multiple dimensions.

### New approaches to discrimination

More recently, authors often implicitly conceptualize discrimination as a process, with some of them identifying causes and consequences of discrimination: “discrimination can have nonprejudicial causes” (Quillian, 2006); “the causes of discrimination are not the same for all types of agent behavior” (Ondrich et al., 1999); or “perceived discrimination has different consequences for advantaged versus disadvantaged groups” (Schmitt et al., 2014). In other examples, scholars explicitly refer to discrimination as a process: “prejudice, stereotypes, and discrimination are complex, multi-determined processes” (Dovidio et al., 2010); discrimination should be “[modeled] as a process rather than a single-point outcome” (Pager and Shepherd, 2008); “discrimination may occur at one stage in a process” (National Research Council, 2004); variables “influence the process that leads to ethnic discrimination” (Fox, 2020); etc.

In addition to understanding discrimination as a process, the third wave of scholarship emphasized the need to analyze it in its multidimensionality, i.e., including different levels of analysis and different perspectives: micro-, meso-, and macro-level causes of discrimination. This also calls for an interdisciplinary approach that extends the study of discrimination beyond the field of social psychology and psychology, which resonates with Allport’s encouragement of “specialists to cross boundaries and borrow methods and insights from neighboring disciplines in the interest of a more adequate understanding of a concrete social problem” [Allport, 1954 (1979)]. Half a decade later, this view is still held. Dovidio (2001) insists that these approaches are not in competition but complementary: they allow for a more comprehensive and complete picture of the phenomenon.

A prominent example of such a comprehensive approach to discrimination is the work of Pager and Shepherd (2008), in which they review how discrimination can be measured. They note that one of the difficulties for scholars in measuring discrimination today is that it is less overt than it was in the pre-Civil Rights era and they classify the methods that make it still possible to do so.

### Modeling the process of discrimination

New approaches to the study of discrimination advocate conceptualizing it as a dynamic process and integrating its multidimensionality by including different levels of analysis. However, to date, no contribution has provided a theoretical framework capable of modelling this complex process in an integrated manner.

Drawing on this claim, I follow Tilly’s line of thinking, which uses mechanism-process based explanations of social phenomena. According to Tilly (2008), a process is composed of distinct mechanisms, which can be seen as sequences or episodes. Describing a process therefore involves reconstructing the logical order of these sequences, i.e., identifying which causes lead to which effects or consequences. Accordingly, the process of racial discrimination developed in this paper follows this logic: it consists of five distinct parts (A–E), and the pathways linking them (represented by arrows) correspond to specific mechanisms connecting distinct concepts.

Moreover, research shows that some mechanisms reinforce each other: for example, responses to discrimination may imply increased ethnic identification among minorities, which may reinforce discrimination because of increased visibility, and disadvantaged economic position due to discrimination may fuel negative prejudice against minorities. Such reinforcing processes, regularly presented as a retroactive loop (Osta and Vasquez, 2021), need to be integrated into the general process of discrimination.

Models of middle-range theories already exist in the literature to allow for empirical testing. For example, models describing the relationship between perceived discrimination and health outcomes with mediators (e.g., stress) and moderators (e.g., ethnic identification) of this relationship are common in social psychology (Cano et al., 2016; Walker et al., 2022; Todorova et al., 2010), while other studies draw models explaining the transition from prejudice to discriminatory behavior (Duckitt, 1992). However, these models explain only one part of the entire process.

More general models that map the entire process from causes of discrimination to consequences for discriminated groups are inconclusive because they either omit several mechanisms or are unintuitive. For instance, Feagin and Eckberg (1980) decompose discrimination into several distinct components (motives, actions, and effects) and differentiate these across institutional and societal levels. They then attempt to reassemble these elements into a schematic representation, correctly noting that this had never been undertaken in prior research. However, their diagram remains rudimentary: it does not establish causal linkages between the identified concepts nor retroactive mechanisms, nor does it clarify how these elements interact across the various analytical levels.

A more recent effort to map the dynamics of discrimination can be found in Williams’ contribution (2023). His theoretical model represents a notable advancement in complexity, incorporating sequential stages, retroactive loops, and multiple levels of analysis. However, despite these improvements, the framework omits several core concepts that are central to understanding discrimination. Moreover, the connections between key concepts—for instance, the progression from structural racism to racial inequality—remain underdeveloped and insufficiently disaggregated, limiting the model’s explanatory power.

In short, convincing models of the general process of discrimination are lacking in the literature, which this paper seeks to address by constructing a unified theory that integrates key concepts and middle-range theories into a model that maps discrimination from causes to consequences.

## A unified theory: from causes to consequences

Definitions of racial discrimination can be either very broad, where all inequality is a consequence of discrimination, or very narrow, where discrimination only includes behaviors that intentionally limit a group’s access to equal opportunities (Pettigrew and Taylor, 2015). Definitions can also consider micro- to macro-level dynamics, from interpersonal actions to structural factors, and from direct to indirect forms (Pettigrew, 2015). A common denominator is the existence of *differential treatment* and the assumption of targeted *groups* (Quillian, 2006). This also presupposes the social construction of group boundaries, which are created through social categorization and sometimes lead to intergroup conflicts, more precisely between in-groups and out-groups (Allport, 1954 (1979), Tajfel and Turner, 1979, 1986).

This paper adopts an intermediate and fairly common working definition, close to that used by national and international agencies (National Research Council, 2004): *Racial discrimination can be defined as any form of differential treatment by individuals or institutions based on actual or perceived group membership that disadvantages members of the targeted group.* As authors of the third wave of scholarship on discrimination emphasize, it constitutes a process with causes and consequences. This process is modeled in Figure 2 and is developed step by step in the following sections, from part (A) to part (E) (Figure 2).

### The micro-, meso-, and macro-level causes of racial discrimination (A)

Various causes explain why racial discrimination occurs, and they can be classified into three broad categories of factors presented in part (A) of Figure 2: individual (micro), institutional (meso) and societal (macro) factors. Here I will outline the main theories that structure the field of intergroup relations in this regard.

#### Micro-level factors

At the micro level, social psychologists and economists discuss individual factors (A) that might predispose individuals not only to hold racial prejudice (B) which can (but does not necessarily) translate into discriminatory behavior against minority members (C), but also to discriminate in the absence of prejudice. The former focus on cognitive processes and personality traits, whereas the latter emphasize rational decision-making.

Regarding cognitive processes, it is assumed that a natural and common cognitive process of the human mind is to categorize objects. This process is explained by the “principle of least effort,” as a natural propensity of the brain to seek an “economy of thoughts” to apprehend the world [Allport, 1954 (1979)]. This process of categorization, when applied to human beings, is called “social categorization” and leads to the construction of an in-group, that is “any cluster of people who can use the term ‘we’ with the same significance” [Allport, 1954 (1979)], and out-groups, which are groups that the individual does not identify with. While the social construction of in-group membership is natural, it does not necessarily entail hostility toward an out-group. When hostility does arise, it may be a consequence of various mechanisms (e.g., a response to perceived threat or compliance with social norms), but it is also an inherent feature of social categorization: “the mere perception of belonging to two different groups—that is, social categorization per se—is sufficient to elicit intergroup discrimination in favor of the ingroup” (Tajfel and Turner, 1979). This tendency to value the in-group over the out-group, called in-group favoritism, leads to prejudices that seek to justify such favoritism. If the out-group is lower on the social hierarchy, it may be referred to as a “minority.”

In terms of personality traits, a stream of research initiated after World War II by Adorno and his colleagues (1950) argued that individuals with an authoritarian personality, mostly characterized by uncritical submissive attitude towards established authorities and rigid adherence to conventional norms are particularly prone to prejudice and hostile to minorities because they tend to perceive those who deviate from the norms as reprehensible. Although this theory was later criticized for methodological and conceptual reasons, it stimulated a body of research that broadened the study of personality and prejudice (Chen and Palmer, 2018; Duckitt, 2015; Sibley and Duckitt, 2008; Altemeyer, 1981). More recent research has further expanded this perspective by showing that prejudice is associated with broader personality traits. Among the Big Five (five dimensions of personality), low agreeableness, that is lower empathy, compassion, and concern for others, is the most consistent predictor of generalized prejudice (Crawford and Brandt, 2019). Likewise, Koehn et al. (2019) have showed in their review on Dark Triad research, that Machiavellianism, a trait characterized by manipulativeness, strategic exploitation of others, and a self-interested orientation toward interpersonal relations, has also been associated with greater prejudicial attitudes.

In economics, two major individual-level theories explain discriminatory behaviors. First, taste-based discrimination, introduced by Becker (1971), posits that individuals (e.g., employers, employees, or customers) engage in discrimination because they have a preference against interacting with certain groups and are willing to incur a financial cost to avoid them. Second, statistical discrimination, as described by Phelps (1972), occurs when decision-makers try to minimize the cost in resources of acquiring information about applicants by using stereotypes as a proxy (informational shortcuts). This mechanism still remains subject of empirical studies (Lang and Spitzer, 2020). Both theories assume that discriminatory behavior is the result of conscious decision-making, an idea that has been challenged by psychologists and behavioral economists, who highlight the role of implicit bias (Bertrand et al., 2005).

Finally, other individual-level mechanisms may account for discrimination independently of individual racial prejudice. These mechanisms include behavior shaped by social pressure (advantaging certain groups over others when socially expected or normalized) and interest-based behavior that are instrumental (maintaining one’s advantages and resources) rather than attitudinal. Already in 1948, Merton built a typology including the “unprejudiced discriminator” (Merton, 1948).

In short, individual factors, whether natural cognitive processes, personality traits, or cost/benefit calculations, can cause racial discrimination. They may predispose individuals to hold racial prejudice (B), which may translate into behaviors that disadvantage members of out-groups (C), or they may directly incline people to discriminate without being prejudiced [direct pathway from (A) to (C)], for instance by conforming to norms or perceiving personal advantages in doing so. However, sociologists critique these models for overlooking institutional (meso-level) and structural (macro-level) discrimination, which shape disparities beyond individual choices (Small and Pager, 2020).

#### Meso-level factors

Individuals from minority groups may experience disadvantage not only through direct interpersonal interactions but also through broader societal structures that, over time, have systematically privileged certain groups over others. Institutions such as schools, housing systems, labor markets, and citizenship regimes function as organizational settings where discrimination is both enacted and sustained. These institutions form a critical meso-level link between individual-level (micro) experiences and broader societal (macro) determinants (Fibbi et al., 2021).

Organizational or institutional discrimination refers to policies and recurring practices that perpetuate inequalities based on racialized categories such as ethnicities, religion, or gender. To paraphrase Tilly (1998), social inequalities are embedded in institutional arrangements that use categorical distinctions to structure access to resources and opportunities. In this view, organizational cultures generate and normalize patterns that systematically exclude non-dominant groups.

Institutional discrimination can stem from either intentional or unintentional practices. Intentional organizational discrimination is relatively rare today, as it is widely prohibited by law. Historical examples include apartheid in South Africa, Jim Crow laws in the United States, or exclusionary policies targeting Protestants in Catholic-majority European countries. As for contemporary examples, we can think of anti-hijab laws that have proliferated in Europe (Mancini, 2018) or the segregation of Roma children in Slovakian educational system (European Commission, 2023).

Unintentional institutional discrimination arises from policies that appear neutral but inadvertently disadvantage specific groups, and are therefore unrelated to racial prejudice. For example, requiring certain university degrees for job eligibility may favor individuals from privileged backgrounds who have greater access to higher education (Ward and Rivera, 2014). Similarly, the widespread use of employee referral systems in hiring processes can reinforce existing social homogeneity by privileging candidates within existing social networks, to the detriment of people with migration backgrounds (Elliott, 2001).

To sum up, the institutional dimension of discrimination was neglected in early research but recent scholarship has increasingly emphasized the importance of analyzing it as a distinct and influential mechanism (Mugglin et al., 2022; Fibbi et al., 2021) where institutional settings (A) can cause structural discrimination against members of minorities (C).

#### Macro-level factors

Finally, at the societal level, the structure of society and historical legacies may generate and sustain disadvantages affecting racialized groups. Societal approaches emphasize that discrimination cannot be explained by individual factors alone and have become increasingly prominent in intergroup relations research since the late 1950s (Strabac and Listhaug, 2008). Within this perspective, discrimination is understood as arising in part from social norms (1) and from competition over material or symbolic resources (2).

1. Norms can be enacted in legislation, government policies, court decisions, or media/political discourse, and may directly produce unfair treatment against racialized groups (pathway A–C). In some cases, such norms constitute the vestiges of past discriminatory regimes, through which earlier forms of discrimination continue to shape present outcomes. They can also shape attitudes towards certain groups, as studies and experiments have shown (Ofosu et al., 2019; Shaver et al., 2017; Stangor et al., 2001), thereby fueling racial prejudice that may in turn result in discriminatory practices (pathway A-B-C). Allport already raised the issue by suggesting that stereotypes “are socially supported, continually revived andhammered in, by our media of mass communication—by novels, short stories, newspaper items, movies, stage, radio, and television” (Allport, 1954 (1979)), a view that has initiated a number of theories on media-induced prejudice (Mutz and Goldman, 2010). Social norms not only shape prejudice but also cause structural discrimination (Burns, 2008), even where discrimination is formally prohibited, by legitimizing or justifying unequal treatment through ideologies (racism, xenophobia, Islamophobia, anti-Semitism, anti-black racism, sexism, etc.).
2. Regarding competition, it can be real, as the Realistic Group Conflict Theory with its materialistic assumptions posits (Campbell, 1965), or it can be perceived because of the positioning of the dominant group in the social structure, as the Perceived Group Threat Theory posits (Blumer, 1958). In both cases, discrimination may yield tangible and symbolic gains for dominant groups, such as preferential access to jobs, housing, and political power, as well as the maintenance of social status, group boundaries, and a sense of relative advantage over out-groups.

The perception of threat can be more or less intense depending on various conditions, the most commonly discussed being the economic situation (Blalock, 1967), the relative size of the out-group (Blalock, 1967), and the frequency with which groups are in contact with each other under certain conditions (Stephan and Stephan, 1985, Allport, 1954 (1979)). These theories are often combined into models that account for these different factors and their interactions (Quillian, 1995). Importantly, such competition dynamics not only involve macro-level dimensions, i.e., social hierarchy or size of groups, but also operate at the micro level, for instance in everyday interactions over jobs, housing, or status. Hence, these societal factors may foster the formation of racial prejudice that might materialize in unfair treatments (pathway A-B-C), or directly cause discriminatory actions from majority members perceiving competition (pathway A-C).

To sum up, the micro-, meso-, and macro-level causes of discrimination correspond to part (A) of Figure 2. The way they contribute to the formation of racial prejudice or directly to unfair treatment—whether structural discrimination or discriminatory behavior—are represented by the arrows pointing to parts (B) and (C), respectively. As shown in the figure, these factors may lead directly to structural discrimination or discriminatory behavior without involving prejudice, as indicated by the arrows linking (A) directly to (C).

### Racial prejudice: attitudes and stereotypes (B)

Micro-, meso-, and macro-level factors may contribute to the formation of racial prejudice, which can in turn translate into discriminatory treatment. However, prejudice remains a distinct concept and represents only one possible step in the pathway from multi-level causes to discriminatory behaviors or structures, as prejudice may exist without discrimination and vice versa. Accordingly, pathways involving prejudice are depicted in grey in Figure 2.

Seminal here is the work of Allport, who defines prejudice as follows: “Ethnic prejudice is an antipathy based upon a faulty and inflexible generalization. It may be felt or expressed. It may be directed toward a group as a whole, or toward an individual because he is a member of that group” [Allport, 1954 (1979)]. This definition embeds the two “essential ingredients” of prejudice: the affective component, expressed in a negative attitude (antipathy); and the cognitive component, expressed in an overgeneralized belief (inflexible generalization) about characteristics ascribed to a group, commonly called a stereotype.

This two-part definition is now widely used in the field. Allport also emphasized that overgeneralization and prejudice are the results of normal brain processes: “Why do human beings slip so easily into ethnic prejudice? They do so because the “two essential ingredients [of prejudice]—*erroneous generalization* and *hostility*—are natural and common capacities of the human mind” [Allport, 1954 (1979)].^2^ Subsequent theoretical developments stemming from research on implicit cognition (Greenwald and Banaji, 1995) have refined this definition by including the implicit (unconscious) and explicit (conscious) nature of prejudice (Gaertner and Dovidio, 1986): attitudes and stereotypes also have implicit modes of expression that remain unknown to the prejudiced person.

Finally, an important concept in the process of discrimination is stigmatization, which is directly related to prejudice. Social stigmas are characteristics that deviate from normative expectations and discredit the individual who possesses them. This individual is then said to be “stigmatized.” As Goffman wrote, “stigma is a special kind of relationship between attribute and stereotype,” where the attribute “that stigmatizes one type of possessor […] is neither creditable nor discreditable as a thing in itself” (Goffman, 1963).^3^

Racial prejudice and its components (attitudes and stereotypes), correspond to part (B) in Figure 2. It may link causes to unfair treatments (A-B-C), but not necessarily (as indicated by grey arrows), since some pathways from multi-level factors to unfair treatment do not involve prejudice (A–C).

### When prejudice translates into unfair treatment (C)

Macro-, meso-, and micro-factors and racial prejudice can materialize in unfair treatment against out-group members, which can be either individual behaviors or organizational processes that disadvantage out-group members. However, there is no inevitable transition from prejudice (attitudes and stereotypes held in people’s minds) to discriminatory actions [“acting-out prejudice”; Allport, 1954 (1979)], and discriminatory actions are not necessarily driven by prejudice. In other words, prejudiced individuals may never discriminate against out-group members, while unprejudiced individuals may discriminate against minority members simply because they conform to norms (Merton, 1948). Moreover, there may even be a discrepancy between support for principles of equal treatment and actual discriminatory actions (Quillian, 2006). Intention to discriminate can also be introduced as an intervening mechanism linking prejudice to discriminatory behavior. In their meta-analysis, Schütz and Six (1996) found that intentions to discriminate are more strongly associated with actual discriminatory behavior than prejudice with discriminatory behavior: discriminatory intentions partially explain how prejudicial attitudes translate into discriminatory acts. In short, prejudice does not inevitably result in discrimination because individuals may refrain from acting on their prejudices due to personal values, social norms, institutional constraints, or anticipated sanctions. Thus, unfair treatment can be defined as behaviors or processes that make distinctions based on individual or group characteristics, correctly or incorrectly attributed, that result in some form of exclusion of the targeted individual or group of individuals.

These unfair treatments can take various forms, including explicit (direct) behavior such as verbal antagonism (from racist jokes to verbal abuse), avoidance, segregation (as any form of exclusion from allocation of resources or access to institutions), physical assault and extermination; statistical discrimination (Phelps, 1972) or profiling; more subtle behaviors, such as implicit (indirect) behaviors, like blaming the minority for its disadvantaged position, or automatic behaviors that may be unconscious (Gaertner and Dovidio, 1986); and finally, structural discrimination^4^. This latter is the result of societal factors and entails “both institutional discrimination based upon norms, rules, regulations, procedures and defined position that determine access to resources, and also a broader cultural discrimination based upon widely shared social paradigms and related systems of categorization that both constructs and devalues the ‘other’”; it “may operate independent of a person’s attitudes and awareness” (Burns, 2008).

Unfair treatment corresponds to part (C) in Figure 2, where structural discrimination is distinct from individual discrimination since it is produced by societal factors and does not necessarily depend on prejudice.

### Effects on targets: life chances, perception, and indirect effects (D)

While observing discriminatory behavior is very difficult since racial discrimination has usually become illegal, hidden, and socially undesirable, the effects on their targets are more easily captured by scientific scrutiny. There can be three types of effects of discriminatory behavior or structutral discrimination: (1) direct effects on life chances (poorer outcomes in employment, health, housing, etc.); (2) the perception of discrimination (when a negative outcome is attributed to discrimination and not to one’s performance); (3) and indirect effects of this perception on outcomes.

(1) As for the direct effect on life chances, social scientists hypothesize that discrimination reduces life chances by producing—or, rather, contributing to—poorer outcomes in different life domains for minority members, this actual discrimination either being perceived or going unnoticed. Studies usually try to capture the direct effect of discrimination, because “[d]iscrimination may occur at one stage in a process (e.g., the labour market) and contribute only a small amount to racial differences in immediate outcomes” (National Research Council, 2004). In fact, disparities between minority and majority groups (“race gap”) can be explained by factors unrelated to direct discrimination such as different socio-demographic features, human capital, or contextual factors. The remaining net disadvantage after accounting for these factors is used as a proxy for direct effect of discrimination (Heath and Cheung, 2007).
  The most studied domains are labor markets, education, housing, credit, consumer markets, and health. Reviews of empirical research in these domains consistently point to racial disparities that can be attributed to discrimination in all of these domains (Paradies et al., 2015; Quillian et al., 2020; Wiggan, 2007) and with no sign of decline over time (Quillian et al., 2017; Quillian and Lee, 2023; Richman et al., 2018). In turn, poorer socioeconomic outcomes can reinforce prejudice against minority groups while also heightening their perceptions of discrimination, creating retroactive loop: for instance, if Black students perform less well in school or if migrants experience higher rates of unemployment, although these disparities are the consequences of racism, they may contribute to prejudice—such as the belief that Black individuals are less intelligent or that migrants are unwilling to work and instead seek to exploit the welfare system.
(2) Regarding the perception of discrimination, attribution theory accounts for the fundamental mechanism, which assumes that individuals causally explain events, and more specifically, the behavior of other individuals. In other words, individuals try to make sense of events or behaviors by attributing understandable causes to them (Kelley, 1973). Crocker et al. (1998) applied these principles to discrimination. Indeed, stigmatized individuals may attribute negative outcomes to either discrimination or personal failings. This two-option explanation is called ambiguous attribution: “Negative outcomes from others could be due to one’s lack of merit, inferior qualifications, poor performance, or other shortcomings. Alternatively, they could be due to prejudice and discrimination based on one’s devalued social identity” (Crocker et al., 1998). In short, a person’s attribution of a negative event or encounter to prejudice aims to explain it as being the result of a bias against their social category (Schmitt et al., 2014).
  Perceived discrimination may result from actual unfair treatment, but it may also be that actual discrimination goes unnoticed; conversely, an individual or group may attribute a negative event to discrimination when in fact there was none. In short, an individual may correctly attribute a negative outcome to discrimination, but may also over- or underestimate discrimination because of constant vigilance (Crosby, 1984) or because of inferential difficulties (Crocker et al., 1998). It is now widely agreed that perceived discrimination cannot be used as a direct indicator of actual discrimination (Diehl et al., 2021).
(3) Finally, the perception of discrimination (rightly or wrongly attributed) can impact health, wellbeing, and performance. Empirical evidence consistently points to an association between perceived discrimination and poorer mental health/wellbeing outcomes, mainly measured by indicators of stress, anxiety, and depression, but also physical health outcomes such as blood pressure, cardiovascular outcomes, or general self-reported health status (for reviews of empirical research, see Krieger, 1999, Schmitt et al., 2014, Williams et al., 2003, Williams and Williams-Morris, 2000, Pascoe and Richman, 2009). These results provide empirical evidence that supports the social stress theory, which argues that certain groups within society are in a disadvantaged social position, which leads to an increased exposure to social sources of stress and less resources with which to cope with stress (Paradies et al., 2015). In terms of performance, perceptions of prejudice among members of stigmatized groups have also been shown to negatively affect educational or neuropsychological performance (Steele, 1997; Steele and Aronson, 1995; Thames et al., 2013). These findings support hypotheses derived from stereotype threat theory, which posits that individuals from stigmatized groups underperform or refrain from applying to certain jobs “when they become hyper-aware that their performance could confirm the very stereotype they wish to avoid” (Thames et al., 2013), creating a mechanism of self-fulfilling prophecy.

Beyond these immediate consequences, both unfair treatment and its perception can generate retroactive loops that reinforce inequality over time. Perceived or actual discrimination leads to underrepresentation in high-status jobs, poorer educational attainment, or even discouragement from applying to certain positions. These negative outcomes can then be misinterpreted by majority groups as evidence of lower ability or effort, thereby fueling prejudice and legitimizing further discriminatory practices. In this way, initial instances of bias do not remain isolated but accumulate across the life course and across generations, creating a cycle in which discrimination both produces and is justified by the very disparities it helps create.

The effects of unfair treatment correspond to part (D) in Figure 2. These effects may take the form of perceived discrimination or diminished life chances outcomes, and the two can influence each other. A retroactive loop where reduced life chances reinforce prejudice is represented by a backward dash-dotted arrow (D-B pathway).

### Responses to unfair treatment (E)

Once unfair treatment is perceived, targets can respond to it. A compelling theory of responses to discrimination comes from the literature on coping strategies. According to this theory, coping can be defined as “constantly changing cognitive and behavioral efforts to manage specific external and/or internal demands that are appraised as taxing or exceeding the resources of the person,” efforts that are distinct from involuntary behavior such as physiological or emotional (Lazarus and Folkman, 1984). These “demands” are stressors, and scholars have found stigma and unfair treatment to be such stressors (see Miller and Kaiser, 2001, for a review).

Miller and Kaiser (2001) have used this theory to develop a two-fold typology of coping strategies where a target can respond through engaging or disengaging strategies, which has since been widely adopted in later research (Berjot and Gillet, 2011). First, engagement coping includes all strategies that confront the stressor, i.e., unfair treatment and/or its perpetrators. This confrontation can take a collective form (seeking support within the group) or an individual form (such as reporting discrimination to a specialized agency or taking the perpetrator to court).

Ethnic identification has been identified as an important group-based coping strategy, explained within the framework of rejection-identification theory. This theory posits that “the generally negative consequences of perceiving oneself as a victim of racial prejudice can be somewhat alleviated by identification with the minority group,” and that this identification “may be the best possible strategy for feeling accepted and enhancing psychological wellbeing” (Branscombe et al., 1999). While the direction of the relationship between identification and discrimination is often debated, statistical tests on longitudinal data point to discrimination as a “trigger” for strengthening ethnic identification and engaging in activism (Cronin et al., 2012; Schmitt et al., 2014). This phenomenon has been primarily studied by Portes and Rumbaut who coined the term “reactive ethnicity” to describe how “direct experience of discrimination triggers a reaction away from things American and toward reinforcement of the original immigrant identities” (Portes and Rumbaut, 2001). They also explain how such experiences can lead to political mobilization and in-group solidarity. In terms of individual responses, scholars identify all sorts of costs that are important in understanding why individuals choose to respond or not. Indeed, as the homo œconomicus model claims, individuals’ perceptions of discrimination make them feel dissatisfied, a feeling that they will then seek to reduce by choosing to complain if the benefits of complaining appear to outweigh the costs (Feagin and Sikes, 1994; Kaiser and Miller, 2001; Kowalski, 1996). For example, individuals may perceive the time, effort, emotional burden, and financial costs of pursuing legal action as exceeding the expected benefits, including formal recognition of as a victim of discrimination and the possibility of compensation.

More recent studies have highlighted other reasons for (not) reporting among discriminated groups, moving beyond a micro-level study of the experience of discrimination: for example, “[c]onsidering repertoires is an essential macro complement to the generally more micro approaches to resilience and responses to stigma” (Lamont et al., 2016). In fact, cultural and institutional factors also play a role in the choice of a mode of response to discrimination (Koenig, 2017): the cultural repertoires available will enable individuals to draw on specific strategies to formulate their responses and access to resources (e.g., organizational capacities, legal provisions and information) will also determine these strategies (Lamont et al., 2016; Witte, 2018; Lindemann and Stolz, 2022). For example, in contexts where anti-discrimination laws are well known and ethno-religious associations provide support, individuals may be more likely to frame an incident as a violation of their rights and seek legal redress. By contrast, in contexts where discrimination is regarded as an unavoidable part of everyday life and information about the legal framework is limited or inaccessible, individuals may instead ignore the incident or refrain from taking formal action.

Second, disengagement coping includes all strategies for avoiding situations in which unfair treatment may occur, whether through physical, cognitive, or social avoidance, as well as strategies for minimizing or ignoring discrimination (Berjot and Gillet, 2011). Goffman describes such strategies in his famous work on stigma, saying that stigmatized individuals have different possibilities to manage social stigma during “mixed contacts,” i.e., encounters between stigmatized people and who he refers to as “normals”^5^, and there is a continuum between complete secrecy and complete information on stigma. Between the two extremes, individuals can resort to either passing strategies by keeping the stigma unnoticed when it is invisible or unknown (discreditable) or covering strategies by making it less apparent or less disturbing for the normals when it is already known (discredited). Finally, withdrawing to back places can be a way of physically avoiding discrimination. Indeed, Goffman claims that three types of places divide the social world for stigmatized individuals, each characterized by a degree of accessibility: forbidden places are simply closed to stigmatized individuals; civil places are accessible, but the stigmatized individuals are in fact disqualified; back places are accessible, and the individual does not have to make any effort to pass or cover (Goffman, 1963). Such strategies have been investigated among minority groups in empirical research. For example, it has been shown that the self-blame strategy—that is blaming oneself for the experienced racial discrimination—is used among Russian immigrants in Germany and that this disengaging strategy mediates the relationship between perceived discrimination and stress (Goreis et al., 2020). Similar results have been found concerning behavioral disengagement—that is giving up trying to deal with experiences of discrimination—among native Hawaiians in the U.S. (Kaholokula et al., 2017). Qualitative research on discrimination against veiled Muslim women in Europe also shows that some of them avoid “civil places” (public spaces, the labor market, or public education) where they experience discrimination and turn instead to “back places” such as Muslim associations and family spheres (Lindemann, 2021).

Finally, responses to structural or interpersonal unfair treatment can affect both socioeconomic disadvantage (D) and prejudice (B) resulting in retroactive loops: for example, members of minorities may refrain from applying for certain positions in anticipation of discrimination, a phenomenon known as “chill factor” (Heath and Martin, 2013), and therefore remain in low-skilled jobs; reactive ethnicity may result in being more visible as a minority member and therefore more vulnerable to discrimination; group-based responses may imply fighting for equality or engaging in intercommunity dialogue, and therefore influencing legislation or majority attitudes; and so on.

Responses to structural and interpersonal unfair treatment correspond to part (E) in Figure 2: they are caused by perceptions of discrimination and can influence (increase or decrease) life chances and racial prejudice, the latter retroactive mechanism being represented by backward dash-dotted arrows.

Taken together, these five parts—namely, the causes of discrimination (A), the possible formation of prejudice and its components (B), the potential translation of prejudice into discriminatory behavior and structural discrimination (C), the effects of unfair treatment on the minority groups (D), and their possible responses to discrimination (E)—make up the complex process of discrimination illustrated in Figure 2.

### The three perspectives involved in the process of discrimination

To further capture the complexity of discrimination processes, I propose that racial discrimination can be studied from three analytical perspectives: a *dominant perspective* focusing on the attitudes and actions of majority groups, a *minority perspective* centered on the experiences and perceptions of minority groups facing discrimination, and an *objective perspective* aimed at identifying discriminatory outcomes independently of actors’ perceptions. While this typology is not explicitly formalized in the literature, it echoes methodological discussions (National Research Council, 2004). Pager and Shepherd’s (2008) review offers the most systematic articulation of these perspectives: different empirical techniques “provide a range of perspectives that can help to inform our understanding of whether, how, and to what degree discrimination matters in the lives of contemporary American racial minorities.” They distinguish between survey-based measures of minorities’ reported experiences of discrimination (corresponding to the minority perspective adopted here); surveys and interviews examining the attitudes and actions of majority-group members toward racial minorities (dominant perspective); and statistical or experimental approaches that assess inequality in outcomes between groups (objective perspective).

More specifically, the dominant perspective encompasses all aspects of discrimination as seen by the majority society, mainly attitudes and stereotypes held by the majority towards minority groups and discriminatory practices. Until the third wave of research on prejudice, research focused mainly on this perspective, with much effort put into understanding why and to what extent people are prejudiced against out-groups (Dovidio, 2001). This type of research typically uses population survey data to measure the distribution of negative attitudes in the majority population, social psychology experiments to measure either implicit or explicit prejudice against members of minority groups, content analysis of material produced by the majority society (such as media discourse or parliamentary debates), and qualitative interviews or focus groups with potential discriminators (Pager and Shepherd, 2008).

The minority perspective, in turn, is concerned with how members of racialized groups individually or collectively experience unfair treatment (Diehl and Liebau, 2017). This experience can be either the way these individuals perceive discrimination or the way they respond to it. This perspective is usually studied through population surveys with items on perceived discrimination, qualitative interviews with members of minorities, or analyses of self-reports of discrimination in legal records and data collected through specialized reporting systems.

Finally, discrimination can be studied “from the outside,” i.e., by measuring differences in outcomes between majority and minority groups and estimating the extent to which these differences can be attributed to discrimination. This is done mainly through statistical analysis of observational data from population surveys or censuses, and through field experiments, usually in the form of fictitious resumes or confederates soliciting hiring employers (Pager and Shepherd, 2008; Quillian et al., 2017; Pager, 2007).

Of course, this typology of perspectives can have blurred boundaries, with studies falling between two types. For example, research that studies the extent to which the majority population believes or justifies that certain groups are discriminated against (Bonilla-Silva, 2017) examines both the majority perspective and perceptions of discrimination. However, it is usually relatively easy to locate a contribution in one of these three perspectives and one of the five parts of the discrimination process.

The three perspectives involved in the process of discrimination are indicated in Figure 2 as follows: the dominant perspective is represented by the solid rectangles, the minority perspective by the dashed rectangles, and the “objective” perspective by the dotted rectangles.

### Wrap up and application of the model

Figure 2 illustrates the process of racial discrimination, which can be explained as follows: multi-level causes, namely individual, institutional and societal factors (A), can foster both structural and interpersonal unfair treatment against racialized groups (C). These factors may also contribute to the formation of racial prejudice against a group (B), composed of attitudes (affective component) and stereotypes (cognitive component), which may or may not materialize in unfair treatments (C) towards stigmatized individuals. Accordingly, both pathways A-B-C and A-C are possible, where the pathway involving prejudice is represented in grey to emphasize that discrimination does not necessarily require prejudice. These unfair treatments, in turn, have different effects (D) on the targets of discrimination. These effects may be the perception of unfair treatment (which may be under- or overestimated, or remain unnoticed) and the consequences on life chances. Such disadvantages can in turn reinforce racial prejudices against the group creating a retroactive loop indicated by backward dash-dotted arrows. Individuals subjected to discrimination may (or may not) respond (E) to unfair treatment, either by engaging with or disengaging from its source. These responses may also affect disadvantages or prejudices, generating additional retroactive loops indicated by backward dash-dotted arrows. The study of discrimination can examine the dominant, minority, and objective perspectives, indicated by solid, dashed, and dotted squares, respectively.

In terms of application, the model has a dual scope: it can guide empirical research while also supporting pedagogy in higher education. First, the model can serve as a heuristic tool for empirical research on racial discrimination. By breaking down the phenomenon into distinct parts, the model offers a roadmap for systematically reviewing the existing literature. A researcher interested in studying the discrimination faced by a stigmatized group could examine what is already known about each stage in the process, while simultaneously identifying where gaps exist. For example, the literature may provide extensive data on prejudice toward this group but very little on how its members respond to unfair treatment in institutional settings. Mapping the state of the art in this way not only highlights blind spots in current research but also helps justify the relevance and originality of new studies.

Also, the model contributes to clarifying the theoretical framework of racial discrimination research. Scholars can use it to situate their research questions within a broader processual understanding of racial discrimination and to specify the mechanisms they wish to investigate. For instance, a study focused on responses to unfair treatment could explore the consequences of these responses (such as reinforcing retroactive loops), their forms (engaged or disengaged strategies), or their causes (e.g., whether direct interpersonal discrimination is more likely to lead to disengaged responses, and under what conditions). In this way, the model enables researchers to ground their hypotheses in a coherent conceptual framework.

In short, in contemporary contexts in which racial categories are constructed and enacted through normative, institutional, and interpersonal settings, such a model provides a framework for analyzing the production of disadvantages for groups defined by ethnic, religious, or cultural characteristics. It enables an integrated approach that simultaneously accounts for different levels of analysis, perspectives on discrimination, and the causal mechanisms underlying unequal treatment. Moreover, the model incorporates retroactive mechanisms that have been empirically documented but remain insufficiently theorized.

Second, this paper also provides a valuable pedagogical resource for teaching at the undergraduate and graduate levels. Teachers can use the model to structure courses around a particular link in the process—such as how perceived discrimination leads to adverse health, psychological, and/or economic outcomes—or to present a comprehensive overview of the empirical research available for each part in the process in a given country. The paper also offers students a curated set of references to founding authors, contemporary works and key concepts in the social sciences of racial discrimination, giving them both a conceptual map and an entry point into the scholarly debate.

## Conclusion

In the social sciences, racial discrimination is often understood as a process, i.e., a phenomenon with causes and consequences: many authors argue for the “importance of modeling discrimination as a process rather than as a single-point outcome” (Pager and Shepherd, 2008). Therefore, it should be possible to map this process, from causes to consequences, with key concepts used in the field. This paper has done just that, building a unified theory of discrimination that links key concepts and middle-range theories that have emerged in the social sciences since Gordon Allport’s *The Nature of Prejudice* (Allport, 1954 (1979)).

Also, racial discrimination must be understood as a process with multiple perspectives. On the one hand, discrimination can be studied from three different perspectives, which we might call the dominant, minority, and objective perspectives: the dominant perspective refers to how prejudices and stereotypes are formed and how they are sometimes translated into discriminatory behavior on the part of the majority population; the minority perspective refers to how discrimination is perceived and experienced by the stigmatized; the outcomes of discrimination are the objectively measured effects on life chances.

Moreover, discrimination can be studied not only from different perspectives, but also at different “points” in a complex process: as the model in Figure 2 illustrates, micro-, meso-, and macro-level factors can foster the formation of prejudice or structural discrimination against minorities. The former may (but do not always) lead to discriminatory behaviors. Interpersonal and structural unfair treatment may be perceived by minority members and have an impact on their life chances. Finally, stigmatized individuals can respond to discrimination by engaging or disengaging strategies. Retroactive processes account for phenomena in which one aspect of discrimination can reinforce or mitigate another (e.g., socioeconomic disadvantage due to discrimination can fuel prejudice against that group).

However, the issue of causality should also be addressed. While it is true that no single factor causes racial prejudice, but rather a variety of factors, it is also true that no single factor acts independently and that not all factors have the same weight. Rather, they interact with each other, and some factors may be better predictors of the formation of ethnic prejudice, depending on the socio-historical context (Adorno et al., 1950). This is also true of other elements in the process, such as life chances: socioeconomic outcomes are reasonably influenced not only by discrimination itself, but also by the anticipation of discrimination or the strategies used to deal with it. While testing causality represents an empirical challenge, causal mechanisms can be mapped for theoretical purposes.

This unified model of racial discrimination serves multiple purposes. As a heuristic tool, it enables researchers to identify gaps in the study of discrimination against stigmatized groups, to situate their own contributions within the broader process and to produce hypotheses from a comprehensive conceptual framework. It is also pedagogically valuable, offering a synthetis of key concepts and theories in the study of intergroup relations, as well as a resource for instructors designing courses on the topic. Finally, the model highlights both causal mechanisms and retroactive loops—dimensions that are often acknowledged empirically but seldom theorized in an integrated way.

## Footnotes

1 Here, the concept of prejudice is in focus since it was the case in the founding works of the field of intergroup relation. I will show later how prejudice is one distinct concept that can be factored in the whole process of discrimination that I intend to describe.

2 Italics in original text.

3 For a discussion of the many definitions of social stigma in the literature and its theoretical challenge, see: Link and Phelan (2001). Conceptualizing Stigma. *Annual Review of Sociology,* 27, 363–385.

4 This typology is drawn from the report of the National Research Council National Research Council (2004). *Measuring Racial Discrimination,* Washington, DC, The National Academies Press, ibid. Other national and international reports use very similar typologies (CFR, 2022). Incidents racistes recensés par les centres de conseil. Janvier–décembre, 2021. Bern: humanrights.ch & Commission fédérale contre le racisme (CFR), (FRA, 2017). Second European Union Minorities and Discrimination Survey. Main results. Luxembourg: European Union Agency for Fundamental Rights (FRA). as well as scientific studies (Paradies et al., 2015). Racial Discrimination. *In:* WRIGHT, J. D. (ed.) *International Encyclopedia for the Social and Behavioral Sciences.* Second ed. Oxford: Pergamon, Allen, 2020. *Reconfiguring Islamophobia. A Radical Rethinking of a Contested Concept,* Cham, Switzerland, Palgrave Macmillan.

5 “Normals” are individuals whose social identity coincides with socially established norms. Nowadays, the term “members of the majority/dominant society” is preferred.
